# Experimental Study on Mechanical Properties of the Sandwich Composite Structure Reinforced by Basalt Fiber and Nomex Honeycomb

**DOI:** 10.3390/ma13081870

**Published:** 2020-04-16

**Authors:** Zongwen Li, Jianxun Ma

**Affiliations:** School of Human Settlements and Civil Engineering, Xi’an Jiaotong University, Xi’an 710049, China; majx@xjtu.edu.cn

**Keywords:** sandwich structure, basalt fiber, nomex honeycomb, mechanical properties, failure mode

## Abstract

The new sandwich composite structure formed by basalt fiber resin-based sheets and Nomex honeycomb has the advantages of being lightweight and environmentally friendly, as well as having excellent electromagnetic performance. It has very important application prospects in traditional and emerging fields. In this paper, the mechanical properties of this new sandwich composite structure are studied. The results show that, under the condition of flatwise compression, increasing the height of the honeycomb is conducive to improving the compressive capacity of the structure. However, the height should be controlled within a certain range in case of instability and yield of the honeycomb; under the bending conditions, the bending failure mode of the composite structure has gone through five stages. Owing to the honeycomb manufacturing process, the orientation of the honeycomb also has a great influence on the bending strength of the structure. After further analysis, it is found that basalt fiber sheets contribute the most to the bending stiffness of the structure, and the main role of honeycomb is to provide out-of-plane support. In both cases, the failure of specimens is ductile, and the combined structure still has a small amount of bearing capacity and maintains structural integrity. Research on this new type of composite structural material is very beneficial for promoting the application and development of green and lightweight special functional materials.

## 1. Introduction

**Figure 1:**
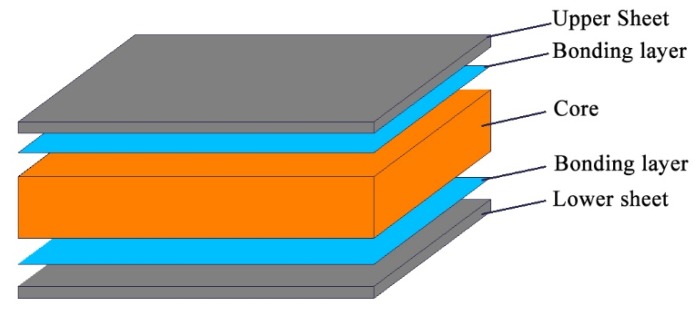
The formation of the sandwich structure.

The sandwich structure plays an important role in modern manufacturing industries such as aerospace and automobile [1,2,3], which can provide higher bending stiffness [4,5] under the same volume density. The Chinese high-speed railway is attracting attention in the world, and this light and high-strength material structure contributes a lot. The sandwich structure is mainly composed of face sheets, a bonding layer, and a core [6], as shown in Figure 1, and it can be divided into the metal sandwich structure and non-metal sandwich structure according to the materials.

With the increasing demand for lightweight and performance-based materials in the structural field, new composite materials such as foam, fiber, and honeycomb have been used more and more widely [7,8]. The representative fibers are glass fiber, carbon fiber, aramid fiber, and basalt fiber; the honeycomb materials include aramid honeycomb, aluminum honeycomb, and so on.

In recent years, owing to cost and environment-friendly factors, basalt fiber gradually stands out among these high-tech fibers, especially in military fields such as radome [9,10,11,12]. Jamshaid H, et al. [13,14] described the green material from stone. V. Dhand, Fiore V, Singha K. A [15,16,17], among others, believe that basalt fiber is an environmental protection natural fiber, which is very suitable to be used as a reinforcement material for manufacturing light and low-cost polymer composite materials, with higher cost-effectiveness and better performance than glass fiber. In addition to environmental protection, basalt fiber has the advantages of good chemical stability (acid and alkali resistance) [18,19] and high-temperature resistance [20], which is more suitable for use in an extreme environment.

Nomex honeycomb is rolled from 100% Meta aramid fiber, which has the advantages of low volume density, high compressive strength, high shear strength, good processability of composite with other materials, and good fatigue strength [21]. Aminanda, et al. [22] studied Nomex honeycomb and aluminum honeycomb, and found that Nomex honeycomb panels have more stable platform stress and better energy absorption performance than aluminum honeycomb panels; Amélie, et al. [23] carried out experimental research on the sandwich structure subjected to medium-speed impact (120 m/s) as aviation armor, and found that Nomex honeycomb panels have lighter weight and higher impact resistance. In the civil industry, such as the Internet, 5G communication, radar antenna, and other military fields, the interference of the metal sandwich structure in electrical performance has seriously affected the development of the industry [24].

Table 1 shows the dielectric constant and dielectric loss of some high-tech fibers [25,26,27]. It can be found that basalt fiber and Nomex honeycomb have excellent electromagnetic properties in addition to mechanical properties. Such materials will be popular among the modern industries that have special requirements, and the composite sandwich panel combined with the two materials will be a new structure with environment-friendly characteristics [28], a stable structure, and good electromagnetic and electrical properties.

Although the new sandwich structure made of basalt fiber resin-based sheet and aramid honeycomb has excellent functional performance, it is not enough to be widely used. Therefore, the research on the mechanical properties of the basalt fiber-Nomex honeycomb sandwich structure is conducive to promoting the application and development of green and lightweight functional materials in special fields.

## 2. Experimental Program

### 2.1. Materials

**Figure 2:**
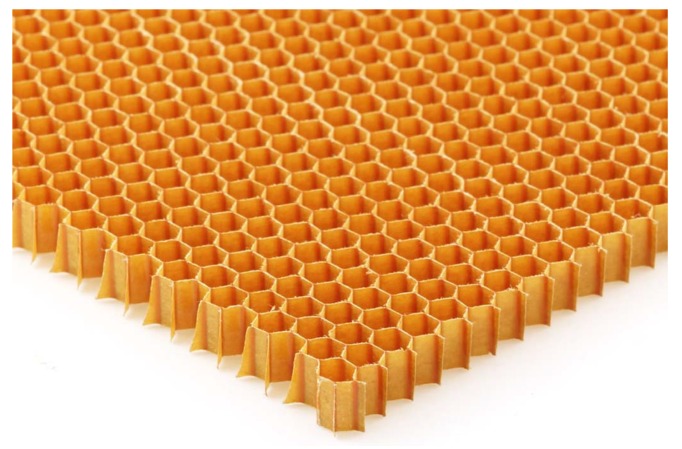
Shape of the honeycomb.

The basalt fiber resin-based plate is provided by Haining Dingqiao Anbang building materials factory (Haining, China), and the length of basalt fiber is 13 μm. The honeycomb is pasted by phenolic resin provided by Easycompositites company (Beijing, China), with a wall thickness of 0.05 mm and an inner diameter of 3.2 mm, as shown in Figure 2. Epoxy resin is chosen as the adhesive, which is provided by Dongguan HeiMa Chemical Co., Ltd., Dongguan, China, which is made up of BH-653 waterborne epoxy emulsion and BH-519 waterborne curing agent; the mixed volume ratio is 2:1. Some mechanical properties of materials are shown in Table 2.

### 2.2. Specimens Preparation

#### 2.2.1. Preparation of Flatwise Compressed Specimens

Under the condition of static compression, the basalt fiber resin-based sheet is assumed as a rigid body without deformation [29], and the thickness of the upper and lower sheet is 1.2 mm. The compressed specimens are only different in the height of honeycomb. The size of each test specimen is shown in Table 3. B is short for Basalt fiber sheet, where B1 means the thickness is 1.2 mm and B2 means the thickness is 1.4 mm. N is short for Nomex honeycomb, where the number after N indicates the height of honeycomb.

#### 2.2.2. Preparation of Bending Specimens

**Figure 3:**
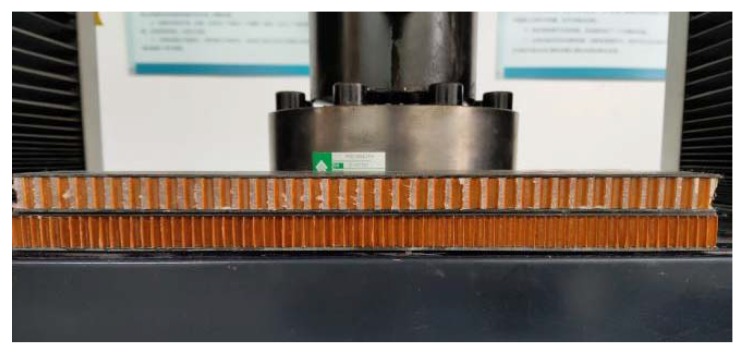
L-shaped specimen (top) and W-shaped specimen (below).

In bending test specimens, the face sheet bears in-plane tension, compression, and in-plane shear stress, and the core material mainly bears transverse shear stress [30,31,32], which is evenly distributed along the thickness. Therefore, the thickness of the basalt fiber and the height of Nomex honeycomb are taken into consideration. Besides, the two sides of the honeycomb in the plane are different. In order to study the bending capacity of two different directions, L-shaped and W-shaped specimens are made in the flexural test experiment, as shown in Figure 3. The parameters of bending test specimens are shown in Table 4.

### 2.3. Experimental Equipment

**Figure 4:**
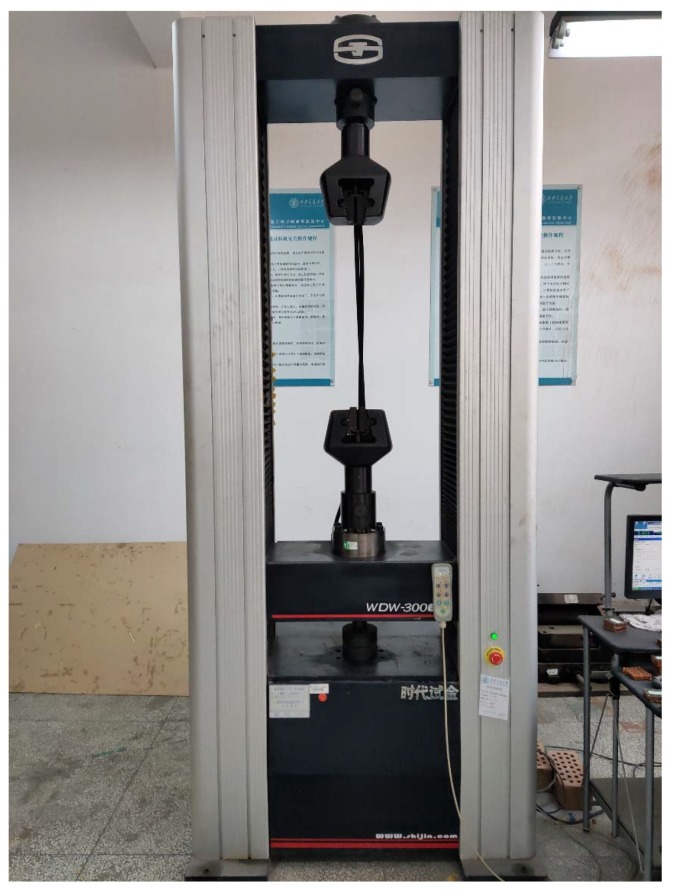
Electronic universal testing machine.

The flatwise compression test and the bending test both use the electronic universal testing machine produced by Jinan Shidai Shijin Corporation, Jinan, China, as shown in Figure 4. The type is WDW-300E, with a maximum loading force of 30 kN. The mechanical dial gauge produced by Gulin Guanglu Corporation (Gulin, China) is used in the bending test; the accuracy value is 0.01 mm.

### 2.4. Experimental Principle and Loading Program

#### 2.4.1. Flatwise Compression Test

**Figure 5:**
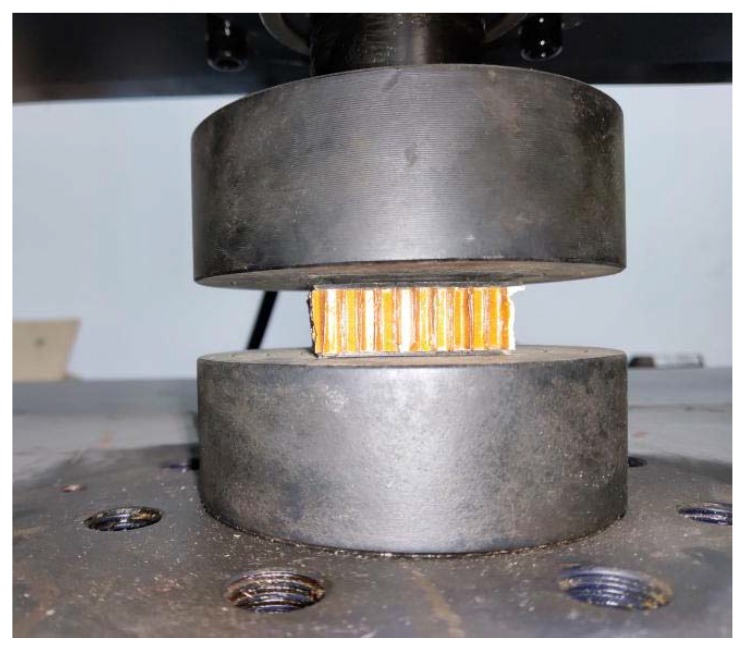
Loading device of the flatwise compression test.

The loading program refers to the test method for flatwise compression properties of sandwich constructions or cores [33,34]. The specimen was placed in the middle between the upper and lower steel pads to make sure the load is perpendicular to the panel, and then adjusted the zero point of the universal testing machine. When testing the modulus of elasticity of flatwise compression, apply the load to about 50% of the failure load according to test method; record the load at all stages and the corresponding deformation value; then apply the load to failure level by level; and record the failure load, failure mode, and flatwise compression strength. The initial loading rate of the indenter in the test is 0.5 mm/min. The loading device is shown in Figure 5.

#### 2.4.2. Bending Test

The loading program refers to the Chinese standard test method for flexural properties of sandwich constructions [35] and American standard test method for core shear properties of sandwich constructions by beam flexure [36].

The test principle is to determine the flexure facing strength, flexure core shear strength, flexure stiffness, and shear stiffness through the overhanging beam three-point bending test of the sandwich structure. The elastic modulus of the structure and the shear modulus of the core can be calculated using the data above.

**Figure 6:**
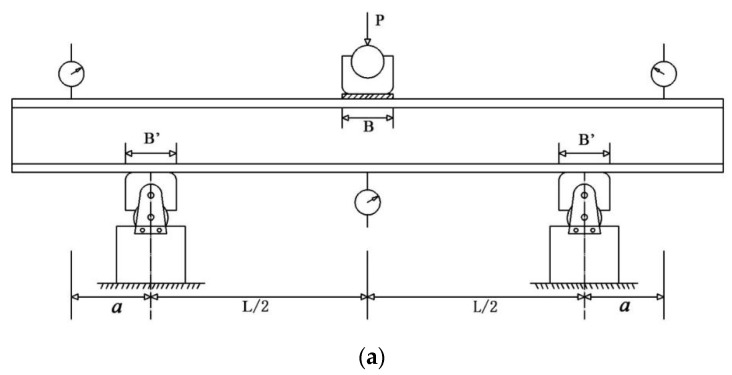

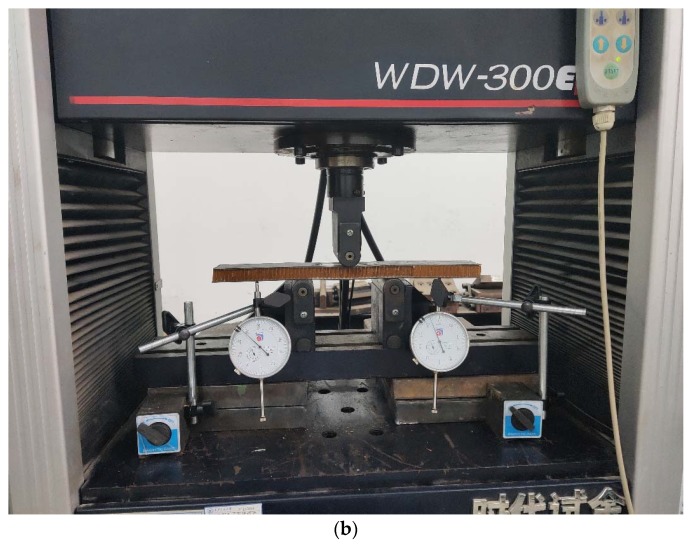
Loading device of the bending test: (**a**) Diagrammatic sketch; (**b**) Test process.

Place the bending specimen in the middle of the upper indenter and the lower two supports, the upper indenter and the support are free rotating cylinders; then, place the mechanical dial gauge (Guilin, China) at the marked points of the overhanging beams on both sides to measure the displacement. After the specimen is fixed, adjust the zero point of the universal testing machine, and the load is applied vertically to the middle of the panel. The initial loading rate of the test indenter is 2 mm/min; the loading device is shown in Figure 6a,b and the parameters of the loading device are shown in Table 5.

## 3. Results and Discussions

### 3.1. Test of Flatwise Compression Properties

#### 3.1.1. Failure Mode

**Figure 7:**
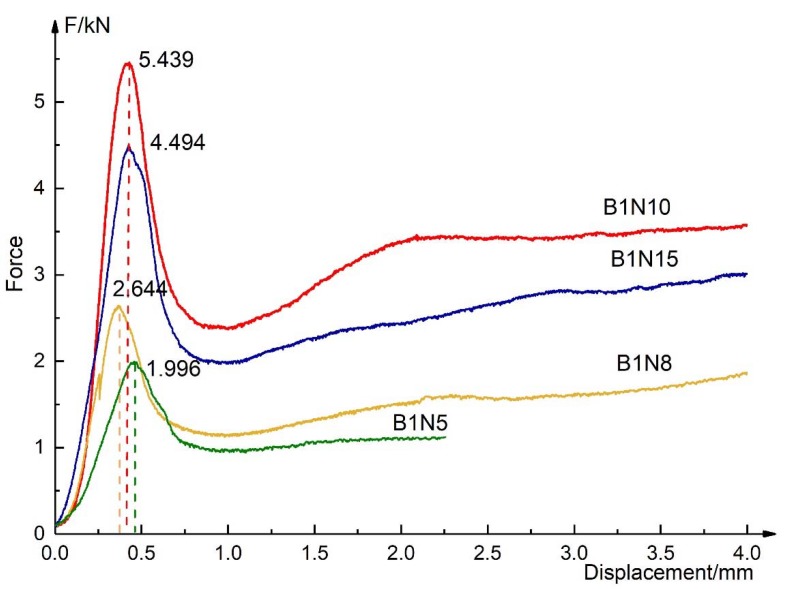
Load–displacement curves of all compression specimens.

Figure 7 shows the load–displacement curves of all compression specimens. It is found that when the core height is 10 mm, the flatwise compression strength is the highest, which is 5.439 kN. The length of the elastic stage is almost the same.

**Figure 8:**
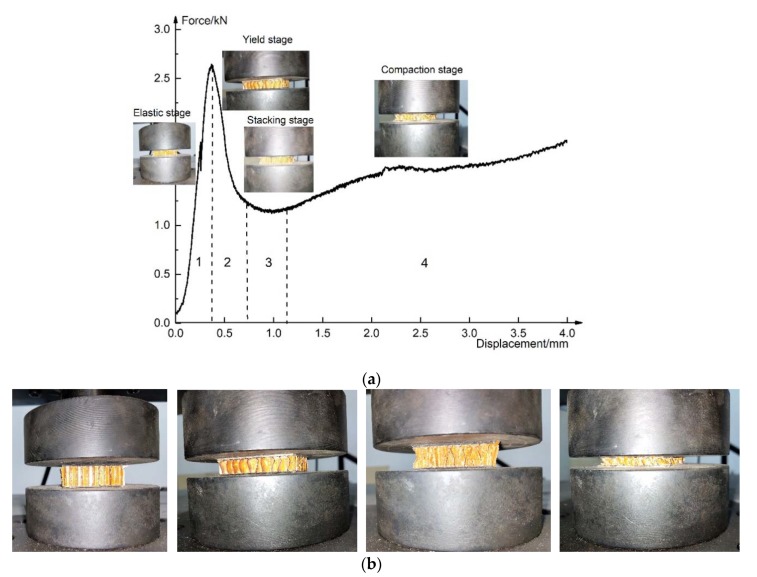
The failure mode and phenomenon of the sandwich structure in the flatwise compression test: (**a**) fail mode; (**b**) phenomenon.

In the process of flatwise compression, it is assumed that the upper and lower sheets are not deformed. According to the load–displacement curve of flatwise compression in Figure 8a, combined with the core deformation process, it is found that the basalt fiber reinforced honeycomb sandwich structure goes through four stages:

(1) The elastic stage; the honeycomb had no change almost, the specimens had no obvious deformation, and a crisp sound was heard in this stage, especially on the specimen B1N15. The sound comes from the damage of the honeycomb wall in the core layer. According to the experimental phenomenon, the damage of the honeycomb wall appeared at the later stage of the elastic stage, especially on the specimen B1N15 because of the larger height and height-thickness ratio of the honeycomb wall.
(2) The yield stage; the honeycomb walls on the four sides of the specimen appeared with wrinkles at first, and the stress growth slowed down.
(3) The stacking stage; the crackling sound of aramid paper extruding was heard from all of the specimens, and the honeycomb was gradually squeezed and compacted from top to bottom; at this stage, the stress began to drop.
(4) The compaction stage; the honeycomb core was completely yielded, with the increase of the indenter’s displacement, the load began to rise slowly. After unloading, the upper sheet rose slowly, and the honeycomb had a spring shape. Although most of the flatwise compression bearing capacity was lost, the specimens’ shape basically remained.

The four stages are shown in Figure 8b.

#### 3.1.2. Flatwise Compression Strength and Elastic Modulus

**Figure 9:**
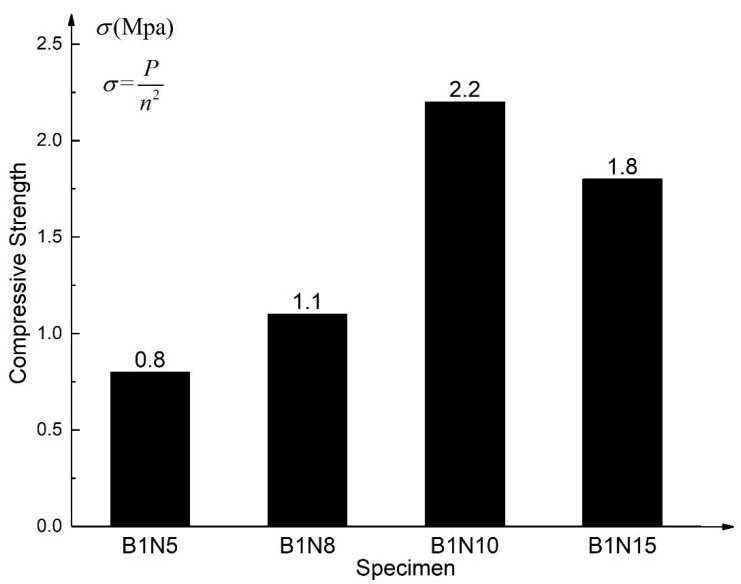
Flatwise compression strength.

**Figure 10:**
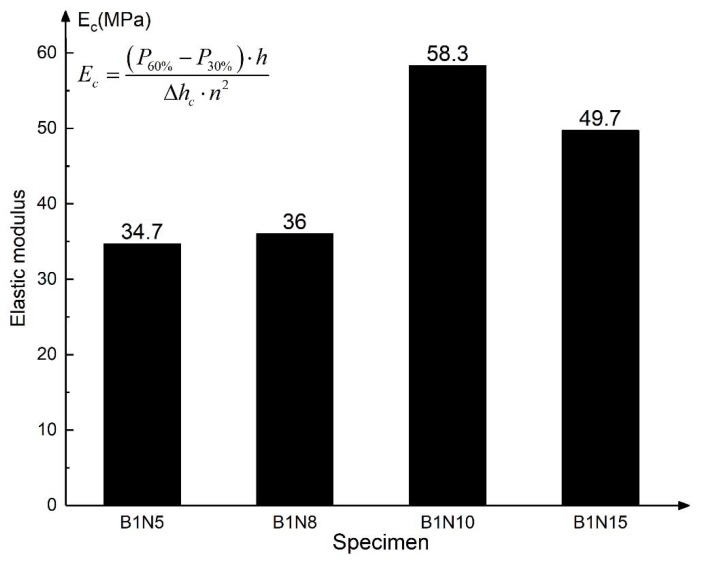
Flatwise compression elastic modulus.

The flatwise compression strength refers to the maximum failure load that the structure can bear under the unit area, which is calculated according to Equation (1). The flatwise compression modulus of elasticity E_c_ of sandwich structure is calculated according to Equation (2) [34]. Choose the 30% failure load and 60% failure load as the starting point and endpoint in the load–displacement curve to calculate E_c_. $\Delta h_{c}$ is the displacement increment relative to the load increment. The flatwise compression strength and elastic modulus are shown in Figure 9 and Figure 10, respectively.

(1) $$\sigma =\frac{P}{n^{2}}$$

(2) $$E_{c}=\frac{\left(P_{60\%}-P_{30\%}\right)\cdot h}{\Delta h_{c}\cdot n^{2}}$$

where *n* is the side length of the test piece and *h* is the height of the honeycomb core.

It can be found that increasing the height of the honeycomb core is conducive to improving the compressive strength of basalt fiber-Nomex honeycomb structure, but when the height of the honeycomb is increased to 15 mm, the compressive strength of the structure decreases owing to the large height to thickness ratio of the honeycomb wall, which is easy to yield.

Comparing the calculated data in Figure 10, the elastic modulus of the structure at the elastic stage also increases with the increasing honeycomb height, but it still decreases when the height is 15 mm. Considered the experimental phenomenon that the honeycomb extrusion sound is particularly obvious during the flatwise compression test of B1N15, it can also be judged that, for the honeycomb with 0.05 mm wall thickness adopted in this test, the structure whose height is 15 mm does not perform well in the flatwise compression test because of the stability problem.

### 3.2. Test of Flexure Properties

**Figure 11:**
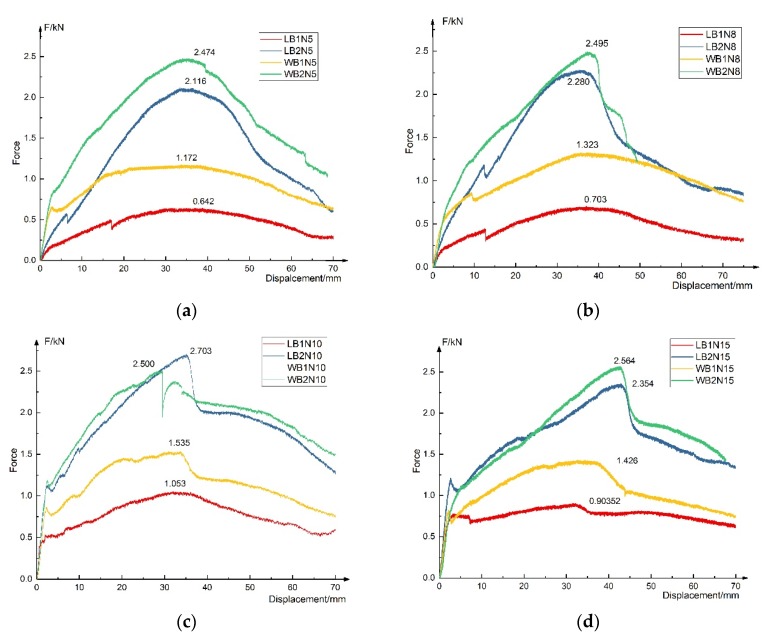
Load–displacement curves of bending specimens: (**a**) Load–displacement curves of N5; (**b**) Load–displacement curves of N8; (**c**) Load–displacement curves of N10; (**d**) Load–displacement curves of N15.

For the sandwich structure, the internal core separates the upper and lower sheet effectively, so that the core increases the section moment of inertia of the structure, and then greatly affects the flexure properties of the sandwich panel [37]. Through the three-point bending test, we can further analyze the failure mode of the sandwich panel and the factors affecting the flexure properties. Figure 11 shows the load–displacement curves of all bending specimens, respectively.

#### 3.2.1. Failure Mode

**Figure 12:**
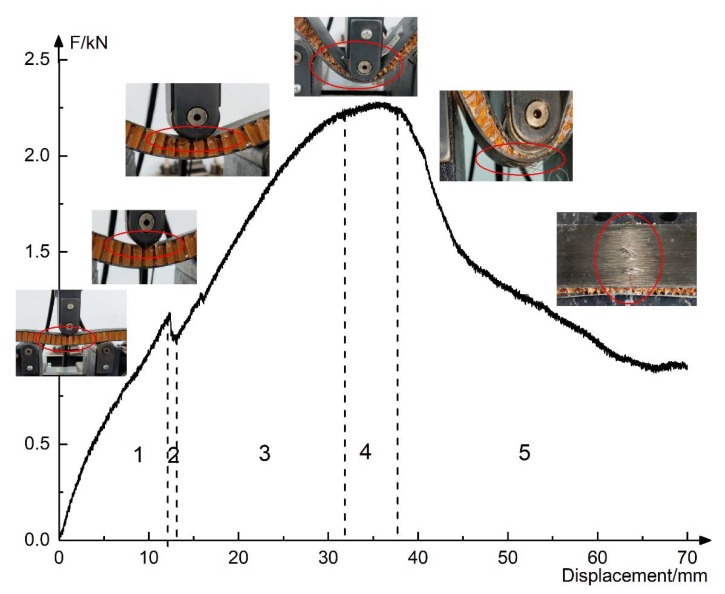
Failure mode of bending specimens.

**Figure 13:**
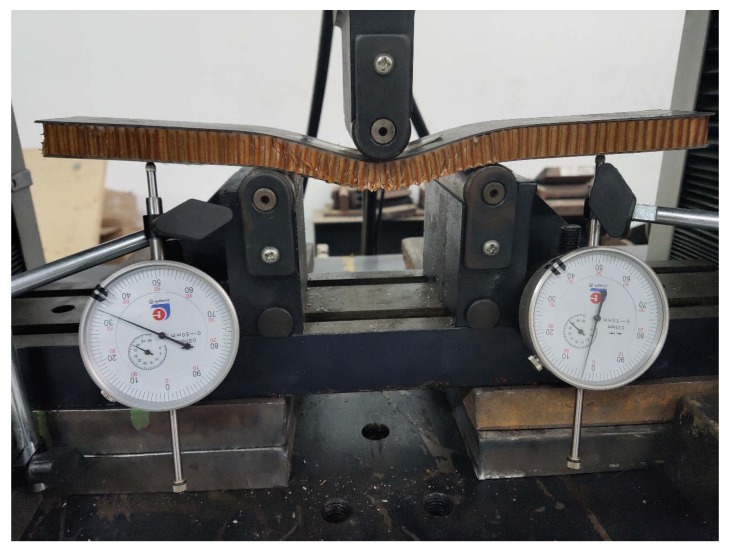
The yielding of the honeycomb cell.

**Figure 14:**
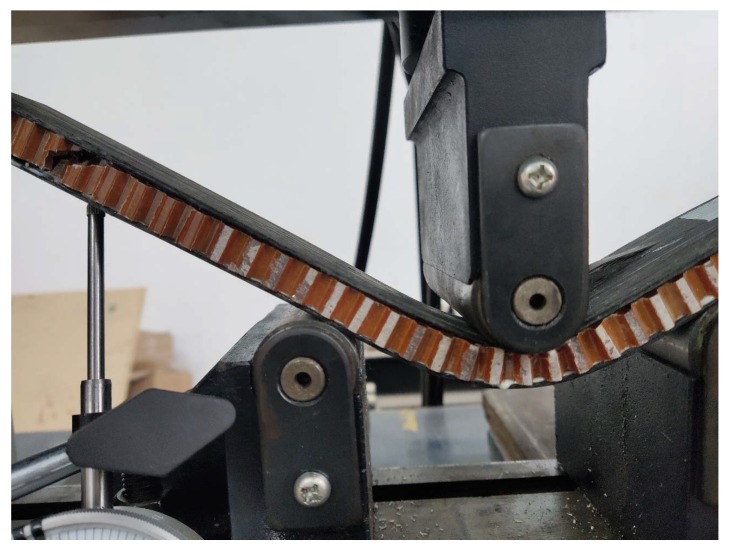
Tear of aramid paper in the bending process.

Figure 12 shows the failure mode of bending specimens and typical experimental phenomenon. The bending process of basalt fiber-Nomex honeycomb structure mainly goes through five processes:

(1) The elastic stage. At this stage, the honeycomb had no obvious deformation.
(2) The yielding stage. With the displacement increases, the load decreased slightly, and fold lines were found on the honeycomb wall, as shown in Figure 12, but the height and the shape of the honeycomb remains unchanged, accompanied by a slight origami sound.
(3) The stacking stage. It was observed that the honeycomb walls were folded; the fold lines spread from bottom to top the middle of the specimens and spread from top to bottom at the support point, as shown in Figure 13. In this stage, the core layer and the sheet may be degummed, and the honeycomb of L-shaped specimens could be torn, resulting in a sudden drop of force, as shown in Figure 14.
(4) The sheet bending resistance stage. In this stage, the honeycomb was folded into a spring shape and lost the out-plane support function to the upper and lower sheet, and then the honeycomb exits from the work and only played the role of connecting sheets.
(5) The sheet broken stage. The resin of the upper sheet was layered and extruded into granules. The basalt fiber of the lower sheet was drawn out, and the crackling sound of the fiber pulling out or breaking was heard. With the increase of displacement, the force dropped sharply and then slowly. Finally, the basalt fiber sheets and the honeycomb were basically out of work, but because of the unbroken resin and the fiber in the sheets, after unloading, the specimens could still recover, and the structure remained complete and remained some strength.

#### 3.2.2. Shear Stress and Flexure Stiffness of the Core

According to the test method for flexural properties of sandwich constructions (GB/T 1456-2005) [36], the shear stress and bending stiffness of each specimen are calculated.

**Figure 15:**
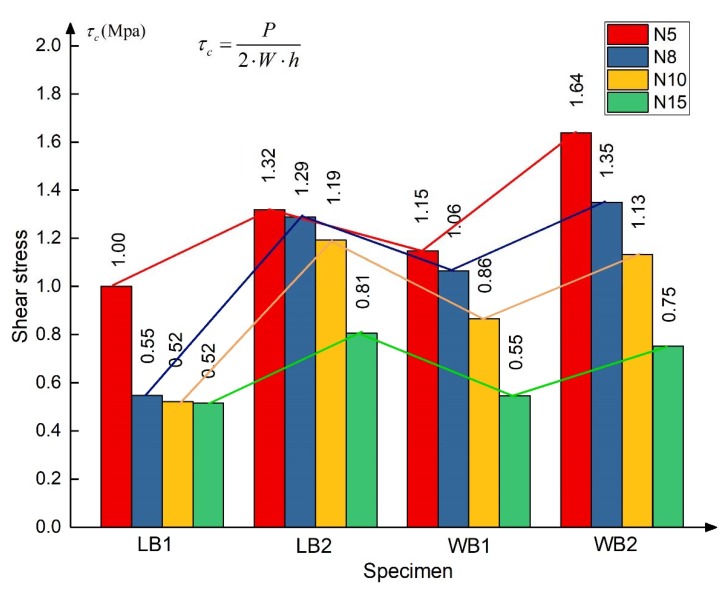
Shear stress of specimens.

(1) In Equation (3), *P* is the mid-span load, *W* is the width of the specimen, *h* is the height of the honeycomb, and the calculation results of each specimen are shown in Figure 15.
  (3) $$\tau_{c}=\frac{P}{2\cdot W\cdot h}$$

There are three points we can get by comparing the shear stress of all testing specimens:

(a) In the specimens with the same direction and height, the shear stress of the core can be effectively increased by increasing the thickness of the fiber sheets.
(b) In the same direction and with the same thickness of fiber sheet, the higher the honeycomb, the smaller the shear stress of honeycomb core, especially for the 15 mm high specimen, as shown in Figure 14. It can be inferred that, the higher the honeycomb, the greater the height thickness ratio of honeycomb, and the more likely the honeycomb wall will yield in the bending process, which will reduce the shear stress of honeycomb core.
(c) The shear stress of the W-shaped specimen is higher than that of the L-shaped specimen with the same sheet thickness and honeycomb height beside the specimen WB2N10; the possible reason is the broken bonding layer between the sheet and core.

**Figure 16:**
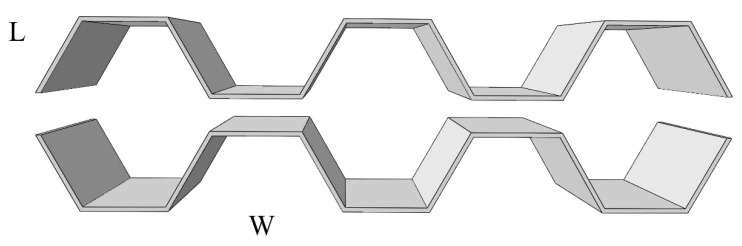
The formation of the honeycomb cell.

The production process of the honeycomb core explains the difference between the W-shape and L-shape. Honeycomb is a long strip of Nomex paper pasted along the L direction, as shown in Figure 16, which leads to the phenomenon that Nomex paper will be pulled off and broken when the core is subject to the shear stress in the L direction, as shown in Figure 14, thus reducing the shear stress.

**Figure 17:**
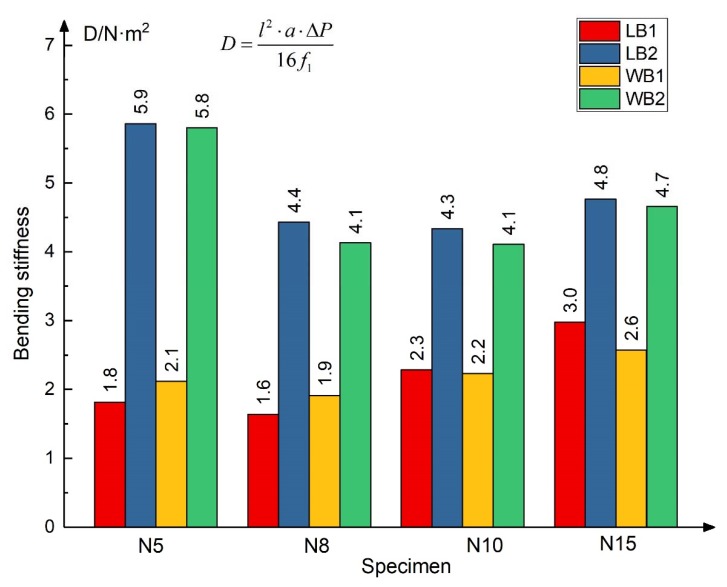
Comparison of the flexure stiffness of bending specimens.

(2) The flexure stiffness of the structure is calculated according to Equation (4), where *l* is the span length, *a* is the overhanging length of the specimen, ΔP is the load increment value of the initial section of the curve, *f*_1_ is the deflection increment value of overhanging point (the average of the left and right points), and the calculation *D* of each specimen is shown in Figure 17.
  (4) $$D=\frac{l^{2}\cdot a\cdot \Delta P}{16\cdot f_{1}}$$

Two points can be found from the analysis of the flexure stiffness.

(a) The honeycomb direction has little influence on the flexure stiffness of the composite structure, and the difference between the flexure stiffness calculated by the specimens (red and yellow, blue and green) is within 15%. It can be considered that the L-type and W-type are at the same level; the possible reason is that the calculation of the flexure stiffness is measured in the elastic stage, and the honeycomb will not be broken at this stage.
(b) The thickness increase of basalt fiber sheet can significantly improve the flexural stiffness of the structure, with a maximum increase of 323%, while changing the height of honeycomb has little effect on the flexural stiffness of the structure.

#### 3.2.3. Influence of Basalt Fiber Sheet Thickness and Honeycomb Orientation

No matter the L-type or W-type, although the thickness of the sheet increased only 0.2 mm from 1.2 to 1.4 mm, the maximum flexure load was increased by more than twofold. Increasing the thickness of the sheets can also improve the flexural modulus of elasticity distinctly.

The bending specimen of the W direction has better performance than that of the L direction, especially when the thickness of the fiber sheet is 1.2 mm.

The displacement corresponding to the peak load of the specimens with the same honeycomb height is almost the same, and the interval length of each stage is almost the same.

The analysis shows that, in the combination of basalt fiber sheet and honeycomb, the honeycomb plays an out of plane supporting role in the structure, and in the elastic stage, the upper and lower basalt fiber sheets contribute more to the improvement of bending stiffness.

## 4. Conclusions

Through the mechanical test of the basalt fiber Nomex honeycomb specimen, the following can be found:

(1) The strength of the compression specimen is greatly affected by the height of honeycomb. For the honeycomb core material with 0.05 mm thickness used in this experiment, the peak value of flat compression strength appears when the height of honeycomb is 10 mm. The high honeycomb core will lead to the premature yield of honeycomb, resulting in the drop of flat compression performance of the composite structure.
(2) Three main factors affect the flexure properties: sheet thickness, honeycomb height, and honeycomb direction. Among them, the sheet thickness has the greatest influence on the flexure properties. The greater the thickness, the greater the flexure strength, the shear strength of the core, and the flexure stiffness of the structure. The influence of the honeycomb height on the flexure properties is complex. The effect on the bending strength of the structure is similar to that of the flatwise compression test, and it reaches the peak value at 10 mm, but it has a negative effect on the shear strength and flexure stiffness of the core. The flexure strength and core shear stress of the W-shaped specimen are greater than that of the L-shaped specimen and have little influence on the flexure stiffness.
(3) No matter the sandwich structure under the flat pressure or bending condition, there is no shape fracture in the loading process. After unloading, the specimen can slowly recover its original shape. The fiber pulling out and fiber fracture of honeycomb crushing and basalt fiberboard can absorb the energy of loading and have good ductility.

## Figures and Tables

**Table 1:** Comparison of dielectric constant and dielectric loss of high-tech fiber.

Parameter	Basalt Fiber	Glass Fiber	Quartz Fiber	Nomex Honeycomb
ε	2.61	4~7	3.78	1.07
tanδ	0.005	0.0026~0.0068	0.0002	0.0028

Note: ε is the dielectric constant; tanδ is the dielectric loss.

**Table 2:** Mechanical properties of materials.

Properties	Basalt Plate	Basalt Fiber	Resin	Honeycomb
Density (g/cm^3^)	1.6	2.80	1.04	0.048
Tensile strength (MPa)	≥1000	3400–4500	78	–
Elastic modulus (GPa)	≥50	88–91	3.65	–
Elongation percentage (%)	2.6	4.7	7.4	–
Flexural strength (MPa)	–	–	146	–
Flexural modulus (GPa)	–	–	3.35	–
Compressive strength (MPa)	–	–	–	1.5–2.5
Shear strength (MPa)	–	–	–	0.5–1.5

**Table 3:** Parameters of compressed test specimens.

Kind	Sheet Size L × W (mm)	Sheet Thickness s (mm)	Core Height h (mm)	Total Thickness H (mm)	Number
B1N5	50 × 50	1.2	5	7.4	3
B1N8	50 × 50	1.2	8	10.4	3
B1N10	50 × 50	1.2	10	12.4	3
B1N15	50 × 50	1.2	15	17.4	3

**Table 4:** Parameters of bending test specimens.

Type	Panel Size W × L (mm)	Sheet Thickness s (mm)	Core Height h (mm)	Total Thickness H (mm)	Number
LB1N5	50 × 350	1.2	5	7.4	3
WB1N5	50 × 350	1.2	5	7.4	3
LB1N10	50 × 350	1.2	10	12.4	3
WB1N10	50 × 350	1.2	10	12.4	3
LB1N8	50 × 350	1.2	8	10.4	3
WB1N8	50 × 350	1.2	8	10.4	3
LB1N15	50 × 350	1.2	15	17.4	3
WB1N15	50 × 350	1.2	15	17.4	3
LB2N5	50 × 350	1.4	5	7.8	3
WB2N5	50 × 350	1.4	5	7.8	3
LB2N10	50 × 350	1.4	10	12.8	3
WB2N10	50 × 350	1.4	10	12.8	3
LB2N8	50 × 350	1.4	8	10.8	3
WB2N8	50 × 350	1.4	8	10.8	3
LB2N15	50 × 350	1.4	15	17.8	3
WB2N15	50 × 350	1.4	15	17.8	3

**Table 5:** Parameter of the bending test loading device.

Span L (mm)	Overhanging Length a (mm)	Width of Indenter B (mm)	Width of Supports B’ (mm)	Width of Specimen W (mm)
120	60	10	10	50
